# Viewpoints on Factors for Successful Employment for Adults with Autism Spectrum Disorder

**DOI:** 10.1371/journal.pone.0139281

**Published:** 2015-10-13

**Authors:** Melissa Scott, Marita Falkmer, Sonya Girdler, Torbjörn Falkmer

**Affiliations:** 1 School of Occupational Therapy and Social Work, Curtin University, Perth, Western Australia, Australia; 2 Cooperative Research Centre for Living with Autism Spectrum Disorders (Autism CRC), Long Pocket, Brisbane, Queensland, Australia; 3 School of Education and Communication, CHILD programme, Institute of Disability Research, Jönköping University, Jönköping, Jönköping County, Sweden; 4 Rehabilitation Medicine, Department of Medicine and Health Science (IMH), Faculty of Health Sciences, Linköping University, Linköping, Östergötland County, Sweden; Lyon Neuroscience Research Center, FRANCE

## Abstract

This article explores the key factors for successful employment from the viewpoints of adults with autism spectrum disorder (ASD) and employers. Two groups of individuals participated in this study, 40 adults with ASD and 35 employers. Q method was used to understand and contrast the viewpoints of the two groups. Data were analysed using by-person varimax rotation factor analysis. Results showed that although both groups appear committed to the employment process, the difference in their understanding regarding the type of workplace support required, job expectations and productivity requirements continues to hinder successful employment. These results highlight the need to facilitate communication between employees and employers to ensure a clear understanding of the needs of both groups are met. The use of an ASD-specific workplace tool may assist in facilitating the necessary communication between these two groups.

## Introduction

Autism spectrum disorders (ASD) represent a category of developmental disorders, characterised by difficulties in social reciprocity, communication and unusual or repetitive behaviour [1]. With a population of 23 million in the working age 15–64 years, it is estimated that there are approximately 115, 400 adults with ASD in Australia [2]. These numbers are expected to increase over the next 10 years. This increase in the number of individuals diagnosed with ASD may partly be due to a change in the diagnostic criteria, resulting in a more accurate and earlier diagnosis [3]. As many adolescents with ASD are now exiting the school system and entering into adulthood, adult services are aware that they are under resourced to manage this period of transition [4].

In Western countries, the labour force participation rate for adults with ASD is 34%, compared with 54% for all individuals with disabilities, and 83% for individuals without disabilities [5, 6]. In fact, individuals with ASD without an intellectual impairment are three times less likely to participate in daytime activities than those with ASD who have an intellectual impairment [6, 7]. For many individuals with ASD interactional difficulties have the largest impact on their ability to apply for and maintain stable employment [8, 9]. This is particularly evident during the interview process as it is the expected requirement of demonstrating social skills and the ‘ability to sell yourself’ that often poses as an obstacle to gaining employment [10]. Once employed social aspects of the work environment are essential to job retention and require employees to continuously engage in social interactions and communication with colleagues [11].

For individuals with ASD it is their ability to manage the social and communication aspects of work [12, 13], rather than their actual job performance that influences their success in finding and maintaining employment [7, 9]. Despite high levels of skills and the desire to work [8], individuals with ASD continue to remain unemployed or underemployed [14]. The consequences of unemployment remains an important issue, as participation in work not only provides the opportunity to earn a livelihood, but is important in identity formation [15]. Work offers a sense of accomplishment and competence, provides structure, and offers an outlet for socialisation and enables people to be full participants in society [15, 16].

The need to support individuals with ASD in obtaining and maintaining employment is widely recognised. To date, most workplace strategies have been impairment focused and have been directed at training for individuals with ASD to overcome their social and communication difficulties [7]. Environmental factors are an essential component in understanding the complex interactions and possible success factors for individual with ASD participating in the workplace [17]. Despite this, there is a paucity of research examining the role of environmental factors in facilitating successful employment of individuals with ASD. Both personal and environmental factors are considered to impact successful employment for adults with ASD [17]. However, it remains unknown how adults with ASD view personal and environmental factors and effect on their workplace success. Hence, the primary aim of the present study was to explore the key factors for successful employment from both the viewpoints of adults with ASD and employers. A secondary aim was to contrast the viewpoints of adults with ASD and employers to explore whether their views on factors for successful employment were similar or different and how these viewpoints impact the process of employment.

## Methods

The Q method provides an in-depth understanding of individuals’ perspectives, attitudes and beliefs regarding a specific topic [18, 19]. In the present study, Q method was utilised to identify, categorise and reveal the viewpoints of adults with ASD and employers on factors for successful employment. The Q method has particular utility in research with adults with ASD, as it allows investigation of viewpoints while reducing the need for verbal communication and social interaction [18].

Q method has the following five phases: 1) developing the ‘concourse’, 2) identifying the Q sort statements, 3) administering the Q sort, 4) factor analysis and 5) interpretation of factors.

### Developing the ‘concourse’

The concourse lays the foundation for the statements regarding employment and was developed through various methods. A thorough search of the literature was conducted through databases: MEDLINE, Scopus, CINAHL, EMBASE, PsycINFO and Web of Science, as well as through a search of ‘grey’ literature. A group of expert researchers in adults with ASD was also consulted to evaluate the face validity of the concourse. The final concourse led to the development of the statements.

### Identifying the Q sort statements

From the concourse, a subset of 91 employment statements was selected and printed on individual paper cards. These statements were piloted with a reference group comprising of 5 adults with ASD, parents of individuals with ASD, teachers with experience in ASD, disability employment co-ordinators, practitioners and researchers. The purpose of the reference group was to select the most relevant and appropriate statements from the original subset of 91 statements. The feedback from the reference group was used to remove statements that were deemed as ‘not relevant’ or difficult to read and comprehend. After the necessary adjustment, a set of 52 statements was selected for the Q-sort pack. The reference group then assessed the Q-sort pack for its readability. Based on the reference group’s final feedback the Q-sort pack was finalised and used in the current study.

### Administering the Q sort

Participants who met the DSM-IV criteria for ASD were invited to take part in the study and were given the opportunity to self-select as having high functioning autism or Asperger’s Syndrome. This group included participants over the age of 18 years, living in Australia, who were currently employed or seeking employment. From this point forward this group of adults with ASD will be referred to as the ‘Employee group’. Participants were excluded from the study if they reported co-morbid conditions distinctly different to the characteristics of ASD that would potentially require additional consideration in successfully attaining employment. The exclusion criteria included: ADHD, epilepsy and psychotic disorders. The employer group included employers who previously have employed and/or are currently employing an adult/s with ASD in their workplace and were accustomed to the employment procedure and management of the workplace environment.

**Fig 1:**
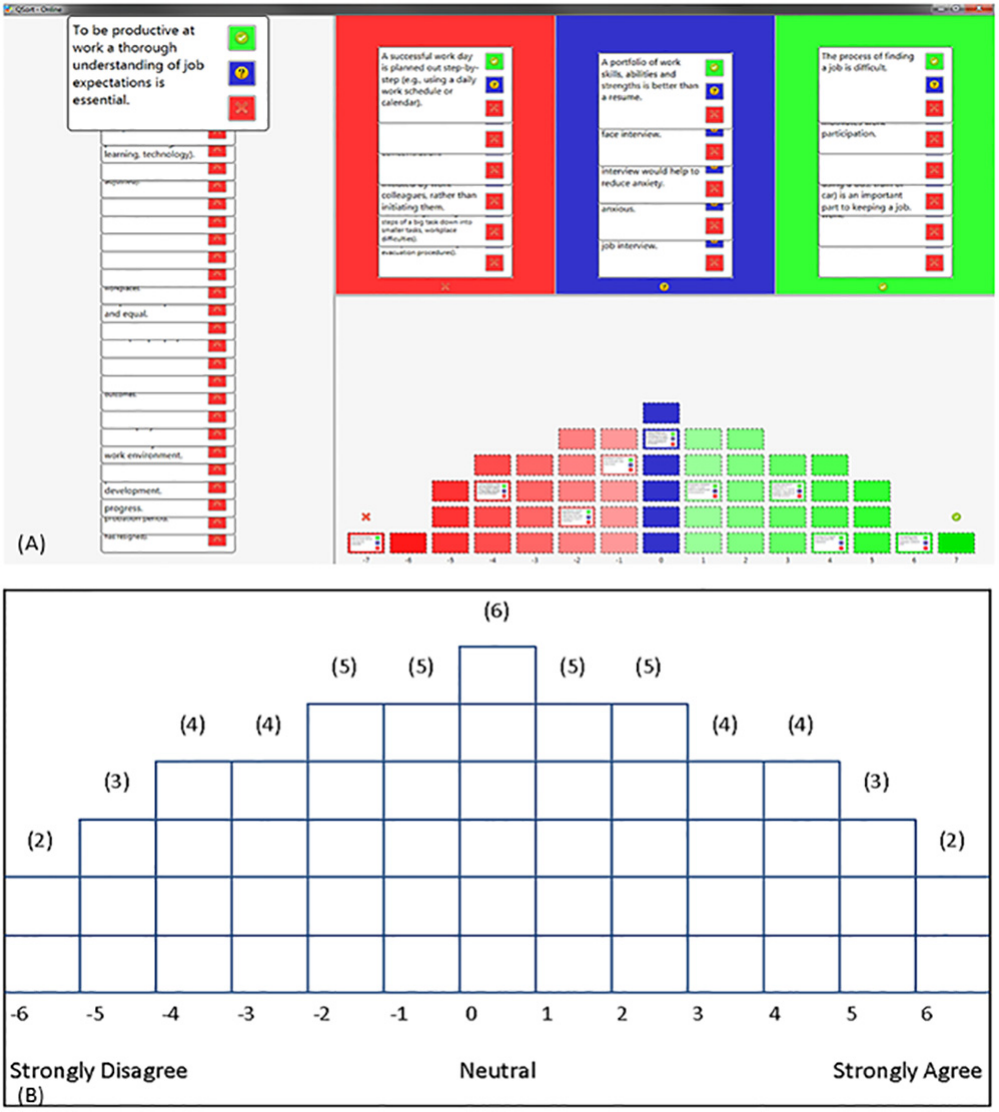
Q sort online and hard copy versions of the sorting grids. (A) Screen shot of participants sorting Q sort statements onto the grid using the online version of the Q sort. (B) Example of the Q sort grid normal distribution.

Participants were recruited across Australia via: the autism associations or organisations within states, universities, disability employment services providers and social media. Information was either posted or emailed to participants explaining the purpose of the study. In total, 75 individuals agreed to participate in this study. The two groups were the employee group (n = 40) and the employer group (n = 35). Participants were presented with the choice of completing the Q sort either online or in-person using the hard copy, as shown in Fig 1. Participants were asked to carefully read through each statement, followed by sorting the 52 statements onto a sorting grid. The sorting grid ranged from ‘strongly agree’ to ‘strongly disagree’ with ranking values of +6 (strongly agree) through 0 (neutral) to -6 (strongly disagree). The sorting grid presented participants with the maximum number of statements allowed for each rank or column as shown in Fig 1. Participants were instructed that they could only place one statement in each block and that each block required a statement for the sorting grid to be complete. Participants were instructed to take their time, that there were no right or wrong answers and that they had the opportunity to re-position the statements on the grid until they were satisfied with the representation of their views. The time taken to complete the Q sort was recorded for both the online version and hard copy version completed in-person.

### Factor analysis

The PQ Method software package was used to analyse the completed Q sorts [20]. The factors, i.e., the most prominent viewpoints were analysed using by-person varimax rotation factor analysis. ‘This analytical method arranges the factors so that the overall rotated solution best accounts and reflects the explained variance’ [21]^(p28)^. Furthermore, the specific Q sorts that significantly define a viewpoint at the p<0.05 level together with the participants who sorted the statements similarly are revealed. A number of consensus statements were also determined. Consensus statements are those in which there are no significant differences between any of the three factors.

The process of extracting factors followed strict decision-making criteria and has been described in order of importance. The first criterion was the “magic number 7”. Seven factors are considered the default number for extraction using the PQ Method software and are recommended as a starting point [20]. This criterion assists with finding the final set of factors that should account for a sizeable portion of study variance [22]. In the present study, eight factors were entered as the default. The Kaiser-Guttman criterion (eigenvalues) was the second criterion used. This follows the rule that only factors with an eigenvalue of 1.00 or above can be selected. All eight factors met this criterion. The third criterion following extraction was to accept those factors which had two or more significant factor loadings and according to Brown’s equation, i.e., significant factor loading = 2.58 x 1÷ (√no. of items in Q set). For this study the significant factor loading was ± 0.36 at the p<0.01 level. Only factors 1, 2, and 3 for both the employee and employer groups satisfied this criterion (22, 23). The next criterion refers to Humphrey’s rule, which considers a factor significant if the multiplication of the two highest loadings (ignoring the positive or negative sign) is greater than twice the standard error. The standard error for this study was 0.28, resulting in factors 1, 2 and 3 fulfilling this criterion. The last criterion was to perform a ‘scree test’. A scree test involves plotting all the eigenvalues in addition to the explained variances in the percentages. The factors to be extracted are those which occur prior to an inflection in the gradient, i.e., prior to the gradient plateauing. After carefully consulting the criteria, it was determined that 3 factors from each group should be included. Three factors accounted for in 53% the explained variance the employee group and 61% of the explained variance in the employer group.

### Ethics

In the present study, an information letter briefly outlining the study was sent to participants and written informed consent was obtained. Data collected from the study were de-identified and securely stored to maintain the confidentiality and privacy of participants. The study was approved through Curtin University Human Research Ethics Committee (HR141/2014) in Perth, Western Australia.

## Results

### Interpretation of factors

In the employee group, the three factors were defined by 37 participants (92%), where only 3 participants did not load significantly on any of the three factors. In the employer group, the three factors were defined by 33 participants (94%), and only 2 participants did not load significantly on any of these factors. Participants’ demographics are presented in Table 1. A list of each statement and the corresponding rankings and z-scores across each factor can be seen in S1 Table. Once the factors for each group were identified, experts in the field were invited to participate in an open group discussion to name each viewpoint.

**Table 1:** Participant demographics.

	Employee n = 40	Employer n = 35
Age (years)		
Mean (SD)	29.1 (10.7)	44.6 (10.4)
Median	26	44
Gender (n)		
Male	24 (60%)	16 (45%)
Female	16 (40%)	19 (54%)
Employment Status (n)		
Employed	30 (75%)	
Unemployed	9 (22%)	
Retired	1 (2%)	
Q sort completion time (min)		
Mean (SD)	31 (31.6)	23.3 (10.6)
Median	20	20
Range	175	50

### Factor interpretation in the employee group

#### Employee viewpoint 1: “I commit to work and work commits to me”

Viewpoint one was defined by 17 participants, as shown in Table 2. Participants in this group included: 11 males and 6 females, with a mean age of 27.5 years ranging between 18 and 45 years. This group of participants placed great importance on commitment to work as supported by statement 11 given the ranking +5; henceforth labelled 11: +5. This employee group revealed that another factor that enhanced their commitment to work and consequently successful employment occurred when participation in their job was valued, encouraged and supported (7: +4; 3:-5; 10:-5; 8:-6). Participants were not necessarily concerned with being effectively matched to a job that promoted their skill set. Instead, these employees sought a workplace with a designated manager capable of providing the necessary workplace support (31: -4; 4: +6). This required a manager to be approachable, effective and direct in communicating and invested in creating an inclusive workplace (16:+6; 5: +5; 15:-6; 14: +5). An inclusive workplace demonstrated commitment to the employee’s participation. Participants wanted to be included in the workplace and support-related decisions (8: -6). Overall, these participants’ life satisfaction increased when regularly working (27: -5).

**Table 2:** Employee Viewpoint One.

Statements	Viewpoints		
	1	2	3
16 It is important that managers are approachable in the workplace	**6**	3	-1
4 Receiving honest feedback on work performance assists with personal and professional development	**6**	3	0
11 Commitment to work is a valuable employee attribute	**5**	4	-3
14 A good manager assists in resolving conflict between employees to help keep the workplace fair and equal	**5**	1	-3
5 Being direct with colleagues is helpful when asking work related questions	**5**	3	-1
26 To be productive at work a thorough understanding of job expectations is essential	**4**	4	-5
21 Job trials help identify areas where more support is needed (e.g., identifying how tasks can be simplified or specifically adjusted)	**4**	0	4
7 A support plan helps to clarify the roles and responsibilities between employees and employers	**4**	0	3
13 Readily available support from an employment co-ordinator is essential to help with difficult work situations	**4**	0	1
19 Making workplace adjustments will not affect employee job performance	**-4**	-3	2
31 Reporting to several different managers, rather than one main manager for work is preferable	**-4**	-5	-1
38 Short, regular breaks during the day interrupt with concentration	**-4**	-1	-5
51 It is not important that employees are motivated by their work	**-4**	-6	-4
3 Regular follow up by an employment co-ordinator during the probation period hinders the work progress	**-5**	-4	-2
27 Working on a regular basis decreases life satisfaction	**-5**	-3	2
10 Ongoing support from an employment co-ordinator limits work performance	**-5**	-4	-1
15 Communication skills (e.g., listening when others are talking, responding and interacting to conversations, body language) are unimportant in most workplace	**-6**	6	3
**8 A support plan for work should only be agreed upon by the employer, not the employee, employment co-ordinator or any colleagues or managers involved**	**-6**	5	1

#### Employee viewpoint 2: “I’m motivated when I have the right job”

Viewpoint 2 was defined by 17 participants, as shown in Table 3. This group of participants included: 10 males and 7 females with a mean age of 29.1 years ranging between 18 and 56 years. Participants in this group valued the independence they gained from being able to work (52:+6), particularly when working in a job matched to their skills and interests (48: +5; 51:-6). This group felt that their motivation and productivity at work was enhanced when they had: the skills and abilities to fulfil their role and a thorough understanding of the workplace culture and job expectations (25:+5; 24:+5, 15:-6). As this group of participants’ confidence increased in their work tasks they required less support, but still valued regular follow up from one, designated manager (28:-5; 3: -4, 31:-5).

**Table 3:** Employee Viewpoint Two.

Statements	Viewpoints		
	1	2	3
52 Being able to work is important for independence	2	**6**	-4
49 Punctuality is important in the workplace	2	**6**	1
25 A good understanding of the workplace culture is important when beginning a new job. i.e., dress code, social etiquette, workplace values and attitudes	3	**5**	-2
24 It is important to have the right skills and abilities to contribute to the needs and productivity requirements of the workplace	0	**5**	-3
48 Job matching employees to their specific interests motivates work participation	3	**5**	4
11 Commitment to work is a valuable employee attribute	5	**4**	-3
26 To be productive at work a thorough understanding of job expectations is essential	4	**4**	-5
46 It would be helpful to research the workplace website before doing a job interview	-2	**4**	4
37 It is OK to choose to be alone during the lunchbreak	1	**4**	3
3 Regular follow up by an employment co-ordinator during the probation period hinders the work progress	-5	**-4**	-2
32 A sudden, unexplained change to the work schedule does not affect an employee’s ability to continue working as per usual (e.g., staff meeting, manager is off sick, work renovations)	-3	**-4**	2
6 Education training on Autism Spectrum Disorders for all employed staff is unnecessary in the work environment	-3	**-4**	-6
10 Ongoing support from an employment co-ordinator limits work performance	-5	**-4**	-1
28 Constant, high level of support from an employment co-ordinator is required, even when an employee’s confidence in work skills increases	-2	**-5**	5
8 A support plan for work should only be agreed upon by the employer, not the employee, employment co-ordinator or any colleagues or managers involved	-6	**-5**	1
31 Reporting to several different managers, rather than one main manager for work is preferable	-4	**-5**	-1
15 Communication skills (e.g., listening when others are talking, responding and interacting to conversations, body language) are unimportant in most workplaces	-6	**-6**	3
51 It is not important that employees are motivated by their work	-4	**-6**	-4

#### Employee viewpoint 3: “I’m confident in a structured work environment”

Viewpoint 3 was defined by 3 participants, as shown in Table 4. Participants in this group included: 2 males and 1 female with a mean age of 20.5 years ranging between 20 and 21 years. These participants felt confident in their work skills, provided they had ongoing and high levels of support in their workplace (28:+5, 2:+5). This support was viewed as important and needed to be structured to include: broken-down tasks, setting work goals and simplifying tasks (40:+6). Participants also viewed job trials as an effective means to demonstrate their work capacity rather than discussing their skills in an interview (22:+5; 43:+4). To this group, the process of finding a job was not difficult, nor did they find being able to work added any significant value to their independence as adults (47:-6; 52:-4).

**Table 4:** Employee Viewpoint Three.

Statements	Viewpoints		
	1	2	3
40 It would be good if an employee could have weekly contact with an employment co-ordinator to discuss his/her work tasks (e.g., breaking the steps of a big task down into smaller tasks, workplace difficulties)	-1	-2	**6**
34 The lighting of the room can affect an employee’s ability to work	-1	-2	**6**
28 Constant, high level of support from an employment co-ordinator is required, even when an employee’s confidence in work skills increases	-2	-5	**5**
2 It is helpful when the support required from an employment co-ordinator is re-assessed and adjusted after the probation period	3	-3	**5**
22 Job trials are helpful to demonstrate specific skills required in a workplace	0	1	**5**
43 Participating in a job trial is better than attending a face-to face interview	-2	0	**4**
46 It would be helpful to research the workplace website before doing a job interview	-2	4	**4**
21 Job trials help identify areas where more support is needed (e.g., identifying how tasks can be simplified or specifically adjusted)	4	0	**4**
48 Job matching employees to their specific interests motivates work participation	3	5	**4**
51 It is not important that employees are motivated by their work	-4	-6	**-4**
39 It is easier to engage in social conversations when topics are initiated by work colleagues, rather than initiating them	-3	2	**-4**
52 Being able to work is important for independence	2	6	**-4**
30 Workplace mentors can assist with daily work issues	2	1	**-4**
26 To be productive at work a thorough understanding of job expectations is essential	4	4	**-5**
29 If required, workplace mentors can give advice on appropriate social behaviour	1	1	**-5**
38 Short, regular breaks during the day interrupt with concentration	-4	-1	**-5**
6 Education training on Autism Spectrum Disorders for all employed staff is unnecessary in the work environment	-3	-4	**-6**
47 The process of finding a job is difficult	2	3	**-6**

### Factor interpretation for employer group

#### Employer viewpoint 1: “We rely on external support”

Viewpoint 1 was defined by 19 participants, as shown in Table 5. Participants in this group included: 8 males and 11 females with a mean age of 43.7 years ranging between 24 and 57 years. This group of employers were open-minded with regard to employing adults with ASD in their workplace as they viewed working as an important factor in increasing life satisfaction (27:-6). However, this group felt more confident employing individuals with ASD when they received ongoing, external support from disability employment service providers (9:+6; 3:-5). The support that employers required from disability employment service providers was: assistance with difficulty work situations, periods of transition in the workplace (such as adjustments in work hours or the designated manager being on annual leave) and carrying out job trials in the workplace to identify areas where more support was needed (13:+5; 21:+4). This group viewed having a designated manager who worked directly with the employee with ASD as well as with the nominated disability employment service provider as a key factor for successful employment (31: -5; 8:-5; 10:-6).

**Table 5:** Employer Viewpoint One.

Statements	Viewpoints		
	1	2	3
1 Increased support is required for employers and employees when significant changes occur in a workplace (e.g., change in job task, adjustment in work hours, manager is on leave or has resigned).	**6**	1	2
9 The development of an individual support plan (i.e., provides clarity on the type, frequency and duration of support required) assists in achieving successful work outcomes.	**6**	1	1
16 It is important that managers are approachable in the workplace	**5**	6	2
14 A good manager assists in resolving conflict between employees to help keep the workplace fair and equal	**5**	6	1
13 Readily available support from an employment co-ordinator is essential to help with difficult work situations	**5**	1	-4
22 Job trials are helpful to demonstrate specific skills required in a workplace	**4**	0	3
5 Being direct with colleagues is helpful when asking work related questions	**4**	0	0
21 Job trials help identify areas where more support is needed (e.g., identifying how tasks can be simplified or specifically adjusted)	**4**	2	-1
4 Receiving honest feedback on work performance assists with personal and professional development	**4**	4	-2
15 Communication skills (e.g., listening when others are talking, responding and interacting to conversations, body language) are unimportant in most workplaces	**-4**	5	-2
6 Education training on Autism Spectrum Disorders for all employed staff is unnecessary in the work environment	**-4**	-4	3
12 Working in a large team (4 or more people) is better than working in a small team (2–3 people)	**-4**	-3	4
51 It is not important that employees are motivated by their work	**-4**	-3	6
31 Reporting to several different managers, rather than one main manager for work is preferable	**-5**	-5	-1
8 A support plan for work should only be agreed upon by the employer, not the employee, employment co-ordinator or any colleagues or managers involved	**-5**	-6	-2
3 Regular follow up by an employment co-ordinator during the probation period hinders the work progress.	**-5**	-4	3
27 Working on a regular basis decreases life satisfaction	**-6**	-6	1
10 Ongoing support from an employment co-ordinator limits work performance	**-6**	-4	0

#### Employer viewpoint 2: “We provide internal support”

Viewpoint 2 was defined by 12 participants, as shown in Table 6. This group of participants included: 5 males and 7 females with a mean age of 40 years ranging between 36 and 44 years. Participants viewed working as important to increasing life satisfaction and independence (27: -6; 52: +5). Participants in this group were not overly reliant on support from an external source instead, they welcomed the opportunity to work with an employee with ASD. This group’s approach was to provide support from within their team. This included: providing on the job-training, explaining the workplace culture and encouraging effective communication skills (41:+5; 25: +4; v15:-5). Team support was reliant on a manager who promoted a fair workplace, provided honest feedback and was approachable (31:-5; 16:+6; 14:+6; 4: +4). Participants viewed training on ASD for colleagues as moderately important (6:-4).

**Table 6:** Employer Viewpoint Two.

****Statements****	****Viewpoints****		
	****1****	****2****	****3****
16 It is important that managers are approachable in the workplace	5	**6**	2
14 A good manager assists in resolving conflict between employees to help keep the workplace fair and equal	5	**6**	1
11 Commitment to work is a valuable employee attribute	3	**5**	2
41 On-the-job training helps with understanding the workplace rules (e.g., start times, finish times, break times, sick leave, holiday leave, and emergencies evacuation procedures).	1	**5**	1
52 Being able to work is important for independence	2	**5**	6
25 A good understanding of the workplace culture is important when beginning a new job. i.e., dress code, social etiquette, workplace values and attitudes	2	**4**	3
49 Punctuality is important in the workplace	1	**4**	2
24 It is important to have the right skills and abilities to contribute to the needs and productivity requirements of the workplace	2	**4**	-2
4 Receiving honest feedback on work performance assists with personal and professional development	4	**4**	-2
32 A sudden, unexplained change to the work schedule does not affect an employee’s ability to continue working as per usual (e.g., staff meeting, manager is off sick, work renovations)	-3	**-4**	0
10 Ongoing support from an employment co-ordinator limits work performance	-6	**-4**	0
6 Education training on Autism Spectrum Disorders for all employed staff is unnecessary in the work environment	-4	**-4**	3
3 Regular follow up by an employment co-ordinator during the probation period hinders the work progress	-5	**-4**	3
15 Communication skills (e.g., listening when others are talking, responding and interacting to conversations, body language) are unimportant in most workplaces	-4	**-5**	-2
31 Reporting to several different managers, rather than one main manager for work is preferable	-5	**-5**	-1
28 Constant, high level of support from an employment co-ordinator is required, even when an employee’s confidence in work skills increases	-3	**-5**	4
27 Working on a regular basis decreases life satisfaction	-6	**-6**	1
8 A support plan for work should only be agreed upon by the employer, not the employee, employment co-ordinator or any colleagues or managers involved	-5	**-6**	-2

#### Employer viewpoint 3: “We give the opportunity, you work it out”

Viewpoint 3 was defined by 2 participants, as shown in Table 7. This group of participants included: 1 male and 1 female with a mean age of 52.5 years ranging between 50 and 55 years. Participants in this group viewed work an important factor for independence (52: +6). This group was willing to provide job opportunities, however required employees to ensure their inclusion in the workplace. This group’s expectations of employees included: having an understanding of the job expectations to ensure productivity, having the capacity to work as team and engaging socially with colleagues (26:+5; 12: +4; 39: -4; 37: -6). Participants viewed financial assistance and ongoing support from disability employment service providers as helpful when making workplace adjustments (18: +5; 17: +5; 28: +4). This group did not view job matching employees to the workplace and being motivated by their work as particularly important (48: -4).

**Table 7:** Employer Viewpoint Three.

****Statements****	****Viewpoints****		
	****1****	****2****	****3****
52 Being able to work is important for independence	2	5	**6**
51 It is not important that employees are motivated by their work	-4	-3	**6**
26 To be productive at work a thorough understanding of job expectations is essential	3	1	**5**
18 Financial assistance from the Employment Assistance Fund is helpful in allowing workplaces to make workplace adjustments for employees	0	-2	**5**
17 Assistance from an employment co-ordinator is necessary when applying for funding for workplace adjustments	-2	1	**5**
43 Participating in a job trial is better than attending a face-to face interview	0	2	**4**
47 The process of finding a job is difficult	-1	1	**4**
12 Working in a large team (4 or more people) is better than working in a small team (2–3 people)	-4	-3	**4**
28 Constant, high level of support from an employment co-ordinator is required, even when an employee’s confidence in work skills increases	-3	-5	**4**
39 It is easier to engage in social conversations when topics are initiated by work colleagues, rather than initiating them	-2	-2	**-4**
29 If required, workplace mentors can give advice on appropriate social behaviour	1	0	**-4**
48 Job matching employees to their specific interests motivates work participation	2	0	**-4**
33 It would be helpful to use technology to assist with on-the-job learning (e.g., use of the IPad for video modellings, to increase work speed and accuracy, visual prompts or work schedule)	-1	2	**-4**
40 It would be good if an employee could have weekly contact with an employment co-ordinator to discuss his/her work tasks (e.g., breaking the steps of a big task down into smaller tasks, workplace difficulties).	0	-2	**-5**
23 Businesses value a broad range of skills in their employees (e.g., communication, problem-solving, learning, technology)	-1	3	**-5**
30 Workplace mentors can assist with daily work issues	3	0	**-5**
34 The lighting of the room can affect an employee’s ability to work	-1	0	**-6**
37 It is OK to choose to be alone during the lunchbreak	-1	-1	**-6**

### Consensus statements in the employee group

A total of five consensus statements occurred with no statistical significant difference in the scores across all three viewpoints in the employee group. They shared a neutral ranking of zero for item 50, in which all three viewpoints suggested that being able to travel independently to work was not necessarily an essential factor in keeping a job. Participants collectively disagreed that a portfolio was better than a resume (item 42; -2 to -1) and that a copy of the interview questions prior to an interview would assist in reducing anxiety (item 44; 0 to -2). Participants showed a neutrality toward financial assistance being helpful in workplace modification for employees (item 18; 0 to -2). Finally, there was strong disagreement that education training on ASD in the workplace is unnecessary for staff (item 6; -3 to -6).

### Consensus statements in the employer group

There were nine consensus statements with no statistical significant difference in the scores across all three viewpoints in the employer group. They shared a negative ranking for items 19 (disagree; -3) and 39 (-2 to -4) in all three viewpoints. This indicated that employees’ work performance is affected when workplace adjustments are made, and that social engagement is not always made easier when initiated by a work colleague. Participants shared a moderately high positive ranking (1 to 4) regarding the importance of understanding the culture of workplace (item 25) and punctuality (item 49) when beginning a new job. Four items shared moderate neutrality across all viewpoints with rankings moving between slightly disagree to slightly agree. This was evident in items relating to: working in a quite environment (item 35; -2 to 1), that structured planning resulted in a successful work day (item 36; -1 to 2), a portfolio was better than a resume (item 42; -2 to 2) and lastly, that a copy of the interview questions prior to an interview would assist in reducing anxiety (item 44; 0 to -2). A moderately high positive ranking (0 to 4) indicated that employers viewed participating in a work trial as better than a face-to-face interview (item 43).

## Discussion

This study identified three main viewpoints within both the employee and employer Q-sample. In order to better understand these results the *International Classification of Functioning*, *Disability and Health (ICF)* framework was used [23]. The ICF is based on a biopsychosocial perspective of health. It serves as a useful framework to understand functioning and disability and the complex interaction between contextual factors, namely environmental and personal factors, and their impact on adults with autism and employment [17]. The ICF highlights the necessary role that environmental factors play in acting as a facilitator or barrier to participation [24]. Therefore, the ICF was used to categorise the viewpoints on employment of both groups of participants according to the environment, participation or activity, as shown in S1 Fig. According the ICF, the *environment* includes: the physical, social and attitudinal contexts; *participation* is the involvement in a life situation and *activity* is the execution of a task by an individual [23]. Lastly, the ICF was used as a means of contrasting and interpreting the viewpoints between the employee and employer group. This was achieved by mapping the viewpoints for each group onto the ICF framework, as seen in S1 Fig.

Viewpoint one of the employee group, *‘I commit to work and work commits to me ‘*, was categorised as *participation* according the ICF. This suggests that when the employee group is made to feel included in the workplace, whereby their talents and skills are valued and they are actively involved in workplace decisions, their dedication to work is reinforced [7, 8]. This suggests that employees view the inclusiveness of the workplace as an important facilitator to work participation. In contrast, Viewpoint one of the employer group, *‘We rely on external support’*, was categorised as *the environment* according the ICF. Literature reports that one of the factors related to work participation for adults with ASD is having the support of the employer. This includes: modification of the work environment, job adjustments and behavioural management [25]. However, the employer group indicated a lack of confidence implementing workplace modifications without the support and guidance of disability employment organisations [26, 27]. These findings are of interest given that while both the employee and employer groups view support in the workplace as important, the type of support that each group requires differs significantly. The discrepancy in the type of support required by each group may in part account for miscommunication between employees and employers when attempting to create a successful workplace [9].

Viewpoint two of the employee group, *‘I’m motivated to work in the right job’* and the employer group, *‘We provide internal support’* were categorised into *activity* and *participation* respectfully, according the ICF. The employee and employer groups agreed on the importance of work productivity, however their understanding of the job expectations required to achieve this goal of productivity differed [28, 29]. Job expectations are reciprocal and must be considered from both the employee and employer’s perspective. Employees expect responsibility, career advancement, fair pay and job tasks to match their skills and abilities [7, 28]. In exchange, employers expect hard work, loyalty, minimum length of stay and productivity [30]. However, unclear or conflicting expectations between employees and employers are unclear may result in demotivated employees, poor work performance, stress and increased employee turnover [30]. Successful work environments depend on clear descriptions of the specific requirements of the job, a shared understanding of the time in which tasks need to be completed, appropriate training, the necessary resources and a supportive workplace culture [7, 30]. It is likely that work environments which adopt these approaches will create workplaces in which individuals with ASD can excel.

Viewpoint three of the employee group, *‘I’m confident in a structured work environment’*, and the employer group, *‘We give the opportunity*, *you work it out’* differed significantly in their approaches toward successful employment. This is indicated in the categorisation of each group into *the environment* and *activity* respectively according to the ICF. The employee and employer groups agreed on the ease of completing the basic business transaction of recruitment for a job position, which included applying for the job position and hiring for the job position. However, the two groups differed in their approach to the process of maintaining a job [29]. Using the ICF *environmental* component, employees require a supportive, structured and task-adapted work environment to perform their jobs successfully [7, 8, 31]. This suggests that it is the manner in which the environment is structured and modified that influences job retention [32]. In contrast, the employer group presented a different view regarding job retention. This best aligns with the ICF *activity* component in that employers are focused on the execution of job tasks to further business productivity [23]. This suggests that once employers have provided the opportunity to work it is the employee’s responsibility to meet the productivity requirements, in order to maintain their job [10, 28]. The strong differences in viewpoints regarding job retention may explain the difficulty employees have in maintaining a job, which likely results from the lack of available support [25, 27]. As well as, if an employee cannot meet the productivity requirements this may impact and lower the business’s profitability. As a result of lowered productivity employers may find it difficult to retain the jobs of employees who are not performing according to the productivity requirements of the business [8]. This suggests that if job retention is regarded as both the responsibility of the employee and the employer then effective communication regarding productivity in the workplace is required.

This study has revealed that the viewpoints between the employee and employer groups are positioned differently within the framework of the ICF. This difference in the groups’ viewpoints may explain why successfully gaining and maintaining employment for individuals with ASD is a challenge. Although both groups appear committed to the employment process, the difference in their understanding regarding the type of workplace support required, job expectations and productivity requirements continues to hinder successful employment. There is clearly a need for an approach which facilitates communication between employees and employers to bridge the communication gap. This study suggests that an ASD-specific workplace tool is required for employers, which may assist in bridging this communication gap between groups. This tool would also provide employers with essential and practical information to effectively manage and modify the workplace for successful employment outcomes.

### Limitations

It is essential that the Q sort comprises a thorough representation of condensed information relating to ASD and employment [22]. In addition, participants were asked to suggest any areas where information may not have been included in the Q sort. Participants suggested a few areas that should contain more statements. This included: using a visual schedule at work for organisation, having access to a work place mentor, making workplace adjustments and the enjoyment of work. However, the Q sort pack contained statements relating to each of these suggestions (statements 33, 30, 19 and 27, respectively, as seen in S1 Table). Moreover, participants made mention of the need for statements to address workplace bullying, work-related anxiety experiences and disclosure of ASD to an employer and colleagues. This suggests that although the chosen statements were generally representative, there were three areas that will need to be addressed in future studies.

## Conclusion

According to the employee and employer groups, factors for successful employment include: comprehensive job expectations, knowledge of the productivity requirements and support in the workplace to assist in creating an inclusive and modified environment. However, it is the difference of interpretation of each factor that may explain the miscommunication between employees and employers in the workplace and ultimately impact on job retention. This study highlights the need for an approach which facilitates communication between both the employee and employer. The development of an ASD-specific workplace tool may assist in bridging the gap of miscommunication between employees and employers in the workplace as well as assisting with workplace modifications for successful employment outcomes.

## Supporting Information

S1 Table Q Sort statements, factor arrays and *z* scores for each viewpoint in both the employee group (EE) and the employer group (ER). (DOCX)

S1 Fig Interaction between the components of the ICF. This figure is based on the World Health Organization ICF framework.
(TIF)
