# Correction: Viewpoints on Factors for Successful Employment for Adults with Autism Spectrum Disorder

**DOI:** 10.1371/journal.pone.0143674

**Published:** 2015-11-18

**Authors:** Melissa Scott, Marita Falkmer, Sonya Girdler, Torbjörn Falkmer

There are errors in the Funding section. The correct funding information is as follows: The authors acknowledge the financial support of the Bankwest Curtin Economics Centre and the Cooperative Research Centre for Living with Autism Spectrum Disorders (Autism CRC), established and supported under the Australian Government's Cooperative Research Centres Program. The authors acknowledge the financial support of Curtin University to Melissa Scott through the Australian Postgraduate Award Scholarship. The funders had no role in study design, data collection and analysis, decision to publish, or preparation of the manuscript.

There is information missing from the Acknowledgments. The Acknowledgments should read: Our sincere thanks go to Wilson Waters, the software engineer from Alintech. The online version of the Q sort would not have been possible without your development and innovation. We are grateful to all the participants, their families and businesses who participated. A special mention goes to EDGE Employment Solutions, EPIC Employment Service, Barkuma’s Personnel Employment in South Australia, Autism Spectrum Australia and AIM Employment of the Autism Association of Western Australia, for their assistance with participant recruitment in this study.

The authors acknowledge the financial support of the Bankwest Curtin Economics Centre and the Cooperative Research Centre for Living with Autism Spectrum Disorders (Autism CRC), established and supported under the Australian Government's Cooperative Research Centres Program.

## References

[1] Scott M, Falkmer M, Girdler S, Falkmer T (2015) Viewpoints on Factors for Successful Employment for Adults with Autism Spectrum Disorder. PLoS ONE 10(10): e0139281 doi: 10.1371/journal.pone.0139281 26462234 10.1371/journal.pone.0139281 PMC4603894

