# Supplementary material for: Viewpoints on Factors for Successful Employment for Adults with Autism Spectrum Disorder
Source: PLoS One. 2015 Oct 13;10(10):e0139281. doi: 10.1371/journal.pone.0139281 (PMC4603894; doi:10.1371/journal.pone.0139281)
Supplement: S1 Table — (DOCX) [file pone.0139281.s001.docx]

**S1 Table. Q-sort statements, factor arrays and z-scores for each viewpoint in both the employee group (EE) and the employer group (ER).**

| Statements | Viewpoints | | | | | |
| --- | --- | --- | --- | --- | --- | --- |
|  | **EE 1** | **ER 1** | **EE 2** | **ER 2** | **EE 3** | **ER 3** |
|  | **Ranking** | **Ranking** | **Ranking** | **Ranking** | **Ranking** | **Ranking** |
|  | **(z-score)** | **(z-score)** | **(z-score)** | **(z-score)** | **(z-score)** | **(z-score)** |
| 1 Increased support is required for employers and employees when significant changes occur in a workplace (e.g., change in job task, adjustment in work hours, manager is on leave or has resigned) | 1 (0.32) | 6 (1.41)* | -2 (-0.51)* | 1 (0.40) | 2 (0.63) | 2 (0.44) |
| 2 It is helpful when the support required from an employment co-ordinator is re-assessed and adjusted after the probation period | 3 (0.85)ᵃ | 2 (0.86)* | -3(0.77)* | -1 (-0.19) | 5 (1.47)ᵃ | -3 (-0.60) |
| 3 Regular follow up by an employment co-ordinator during the probation period hinders the work progress | -5 (-1.53)* | -5 (-1.67) | -4 (-1.06)ᵃ | -4 (-1.46) | -2 (-0.37)ᵃ | 3 (0.95)* |
| 4 Receiving honest feedback on work performance assists with personal and professional development | 6 (1.67)* | 4 (1.13) | 3 (1.06)* | 4 (0.86) | 0 (0.22)* | -2 (-0.43)* |
| 5 Being direct with colleagues is helpful when asking work related questions | 5 (1.26)ᵃ | 4 (1.17)* | 3 (0.90)ᵃ | 0 (0.19) | -1 (-0.29)* | 0 (0.10) |
| 6 Education training on Autism Spectrum Disorders for all employed staff is unnecessary in the work environmentᵇ | -3 (-1.09) | -4 (-1.58) | -4 (-1.26) | -4 (-1.22) | -6 (-1.83) | 3 (0.68)* |
| 7 A support plan helps to clarify the roles and responsibilities between employees and employers | 4 (1.03) | 3 (1.06)ᵃ | 0 (0.08)* | 3 (0.70) | 3 (0.94) | 0 (0.02) |
| 8 A support plan for work should only be agreed upon by the employer, not the employee, employment co-ordinator or any colleagues or managers involved | -6 (-2.08)* | -5 (-1.64) | -5 (-1.54)* | -6 (-1.99) | 1 (0.48)* | -2 (-0.27)* |
| 9 The development of an individual support plan (i.e., provides clarity on the type, frequency and duration of support required) assists in achieving successful work outcomes | 2 (0.70) | 6 (1.40)* | -1 (-0.09) | 1 (0.45) | 1 (0.36) | 1 (0.19) |
| 10 Ongoing support from an employment co-ordinator limits work performance | -5 (-1.79)* | -6 (-1.77) | -4 (-1.32)* | -4 (-1.48) | -1 (-0.12)* | 0 (-0.10)* |
| 11 Commitment to work is a valuable employee attribute | 5 (1.51) | 3 (0.95) | 4 (1.24) | 5 (1.37)ᵃ | -3 (-0.92)* | 2 (0.37) |
| 12 Working in a large team (4 or more people) is better than working in a small team (2-3 people) | -3 (-0.79) | 4 (-1.10) | -3 (-1.00) | -3 (-1.03) | 0 (-0.02)ᵃ | 4 (1.26)* |
| 13 Readily available support from an employment co-ordinator is essential to help with difficult work situations | 4 (0.96)ᵃ | 5 (1.30)* | 0 (0.03) | 1 (0.40)* | 1 (0.31) | -4 (-1.02)* |
| 14 A good manager assists in resolving conflict between employees to help keep the workplace fair and equal | 5 (1.30)* | 5 (1.34)ᵃ | 1 (0.51)* | 6 (1.81)ᵃ | -3 (-0.88)* | 1 (0.27)* |
| 15 Communication skills (e.g., listening when others are talking, responding and interacting to conversations, body language) are unimportant in most workplaces | -6 (-1.83) | -4 (-1.47) | -6 (-2.11) | -5 (-1.82) | 3 (0.69)* | -2 (-0.54)* |
| 16 It is important that managers are approachable in the workplace | 6 (1.83)* | 5 (1.23)* | 3 (1.01)* | 6 (1.71)* | -1 (-0.18)* | 2 (0.29)* |
| 17 Assistance from an employment co-ordinator is necessary when applying for funding for workplace adjustments | 0 (-0.13) | -2 (-0.86)* | -2 (-0.66)* | -1 (0.07)* | 1 (0.33) | 5 (1.60)* |
| 18 Financial assistance from the Employment Assistance Fund is helpful in allowing workplaces to make workplace adjustments for employeesᵇ | 0 (-0.17) | 0 (0.24)* | -2 (-0.52) | -2 (-0.44)* | 0 (0.07) | 5 (1.35)* |

**S1 Table. Continuing.**

| Statements | Viewpoints | | | | | |
| --- | --- | --- | --- | --- | --- | --- |
|  | **EE 1** | **ER 1** | **EE 2** | **ER 2** | **EE 3** | **ER 3** |
|  | **Ranking** | **Ranking** | **Ranking** | **Ranking** | **Ranking** | **Ranking** |
|  | **(z-score)** | **(z-score)** | **(z-score)** | **(z-score)** | **(z-score)** | **(z-score)** |
| 19 Making workplace adjustments will not affect employee job performanceᵉ | -4 (-1.11) | -3 (-1.04) | -3 (-0.93) | -3 (-1.17) | 2 (0.60)* | -3 (-0.75) |
| 20 Flexibility in the workplace is important when adjusting job tasks | 3 (0.85)* | 1 (0.55) | -1 (-0.16) | 3 (0.67) | 0 (-0.12) | -3 (-0.58)* |
| 21 Job trials help identify areas where more support is needed (e.g., identifying how tasks can be simplified or specifically adjusted) | 4 (1.12) | 4 (1.17)* | 0 (0.04)* | 2 (0.63)ᵃ | 4 (1.06) | -1 (-0.23)ᵃ |
| 22 Job trials are helpful to demonstrate specific skills required in a workplace | 0 (0.29) | 4 (1.19) | 1 (0.30) | 0 (0.28) | 5 (1.31)* | 3 (0.99) |
| 23 Businesses value a broad range of skills in their employees (e.g., communication, problem-solving, learning, technology) | 1 (0.54) | -1 (-0.13)* | 1 (0.55) | 3 (0.71)* | -3 (-0.83)* | -5 (-1.53)* |
| 24 It is important to have the right skills and abilities to contribute to the needs and productivity requirements of the workplace | 0 (0.29)* | 2 (0.71) | 5 (1.34)* | 4 (0.98) | -3 (-0.76)* | -2 (-0.35)* |
| 25 A good understanding of the workplace culture is important when beginning a new job. i.e., dress code, social etiquette, workplace values and attitudesᵉ | 3 (0.93)* | 2 (0.86) | 5 (1.38)* | 4 (1.20) | -2 (-0.32)* | 3 (0.70) |
| 26 To be productive at work a thorough understanding of job expectations is essential | 4 (1.16) | 3 (1.05) | 4 (1.23) | 1 (0.48)* | -5 (-1.62)* | 5 (1.53) |
| 27 Working on a regular basis decreases life satisfaction | -5 (-1.53)* | -6 (-1.79) | -3 (-0.95)* | -6 (-1.85) | 2 (0.57)* | 1 (0.25)* |
| 28 Constant, high level of support from an employment co-ordinator is required, even when an employee’s confidence in work skills increases | -2 (-0.61)* | -3 (-0.87)* | -5 (-1.37)* | -5 (-1.56)* | 5 (1.62)* | 4 (1.08)* |
| 29 If required, workplace mentors can give advice on appropriate social behaviour | 1 (0.47) | 1 (0.29) | 1 (0.19) | 0 (0.21) | -5 (-1.63)* | -4 (-0.83)* |
| 30 Workplace mentors can assist with daily work issues | 2 (0.73)* | 3 (0.99)* | 1 (0.24)* | 0 (0.19)* | -4 (-1.15)* | -5 (-1.87)* |
| 31 Reporting to several different managers, rather than one main manager for work is preferable | -4 (-1.27)* | -5 (-1.63) | -5 (-1.90)* | -5 (-1.76) | -1 (-0.14)* | -1 (-0.19)* |
| 32 A sudden, unexplained change to the work schedule does not affect an employee’s ability to continue working as per usual (e.g., staff meeting, manager is off sick, work renovations) | -3 (-0.93) | -3 (-0.97) | -4 (-1.21) | -4 (-1.27) | 2 (0.52)* | 0 (0.08)* |
| 33 It would be helpful to use technology to assist with on-the-job learning (e.g., use of the IPad for video modellings, to increase work speed and accuracy, visual prompts or work schedule) | -2 (-0.50) | -1 (-0.28)* | -1 (-0.28) | 2 (0.54)* | 3 (0.89)* | -4 (-1.43)* |
| 34 The lighting of the room can affect an employee’s ability to work | -1 (-0.25) | -1 (-0.41)* | -2 (-0.45) | 0 (0.25)* | 6 (1.78)* | -6 (-2.55)* |
| 35 Work environments that are quiet, almost noise free are preferableᵈ | -2 (-0.51)ᵃs | -2 (-0.64) | 2 (0.65) | -2 (-0.57) | 0 (0.10) | 1 (0.16)ᵃ |
| 36 A successful work day is planned out step-by-step (e.g., using a daily work schedule or calendar)ᵈ | -1 (-0.22) | 1(0.35) | 2 (0.60)* | 2 (0.61) | -1 (-022) | -1 (-0.19) |
| 37 It is OK to choose to be alone during the lunchbreak | 1 (0.35) | -1 (-0.29) | 4 (1.09) | -1 (0.06) | 3 (0.71) | -6 (-2.13)* |
| 38 Short, regular breaks during the day interrupt with concentration | -4 (-1.47) | -3 (-0.92) | -1 (-0.27)* | -3 (-0.96) | -5 (-1.80) | 0 (0.06)* |

**S1 Table. Continuing.**

| Statements | Viewpoints | | | | | |
| --- | --- | --- | --- | --- | --- | --- |
|  | **EE 1** | **ER 1** | **EE 2** | **ER 2** | **EE 3** | **ER 3** |
|  | **Ranking** | **Ranking** | **Ranking** | **Ranking** | **Ranking** | **Ranking** |
|  | **(z-score)** | **(z-score)** | **(z-score)** | **(z-score)** | **(z-score)** | **(z-score)** |
| 39 It is easier to engage in social conversations when topics are initiated by work colleagues, rather than initiating themᵈ | -3 (-0.66) | -2 (-0.78) | 2 (0.59)* | -2 (-0.32) | -4 (-1.07) | -4 (-0.91) |
| 40 It would be good if an employee could have weekly contact with an employment co-ordinator to discuss his/her work tasks (e.g., breaking the steps of a big task down into smaller tasks, workplace difficulties) | -1 (-0.21) | 0 (0.23)ᵃ | -2 (-0.47) | -2 (-0.19)ᵃ | 6 (1.93)* | -5 (-1.51)* |
| 41 On-the-job training helps with understanding the workplace rules (e.g., start times, finish times, break times, sick leave, holiday leave, and emergencies evacuation procedures) | 1 (0.55) | 1 (0.61) | 2 (0.70) | 5 (1.26)* | -2 (-0.38)* | 1 (0.17) |
| 42 A portfolio of work skills, abilities and strengths is better than a resumeᶜᵈ | -1 (-0.30) | -2 (-0.45) | -1 (-0.23) | -1 (-0.19) | -2 (-0.65) | 2 (0.31) |
| 43 Participating in a job trial is better than attending a face-to face interviewᵈ | -2 (-0.64)* | 0 (0.25) | 0 (0.10)* | 2 (0.58) | 4 (1.16)* | 4 (1.01) |
| 44 I think that requesting a copy of the interview questions prior to an interview would help to reduce anxietyᵇᵉ | -1 (-0.28) | 0 (-0.06) | 0 (0.02) | -1 (0.17) | -2 (-0.60) | -1 (-0.17) |
| 45 Doing a face to face interview usually makes job applicants anxious | 0 (-0.20)ᵃ | 0 (0.02) | 2 (0.67) | 3 (0.67)* | 2 (0.57) | -2 (-0.35) |
| 46 It would be helpful to research the workplace website before doing a job interview | -2 (-0.35)* | -2 (-0.43) | 4 (1.15) | 2 (0.55) | 4 (1.15) | 0 (0.00) |
| 47 The process of finding a job is difficult | 2 (0.65) | -1 (-0.23)* | 3 (0.76) | 1 (0.45) | -6 (-2.61)* | 4 (1.10) |
| 48 Job matching employees to their specific interests motivates work participation | 3 (0.82) | 2 (0.62) | 5 (1.34) | 0 (0.32) | 4 (1.01) | -4 (-0.83)* |
| 49 Punctuality is important in the workplaceᵈ | 2 (0.69) | 1 (0.52) | 6 (1.63)* | 4 (0.96)* | 1 (0.25) | 2 (0.37) |
| 50 Being able to independently travel to and from work (e.g., using a bus, train or car) is an important part to keeping a jobᶜ | 0 (0.30) | 0 (0.03) | 0 (0.11) | -2 (-0.93)ᵃ | 0 (-0.05) | -1 (-0.12) |
| 51 It is not important that employees are motivated by their work | -4 (-1.53) | -4 (-1.30) | -6 (-2.18)* | -3 (-1.00) | -4 (-1.02) | 6 (1.80)* |
| 52 Being able to work is important for independence | 2 (0.82)* | 2 (0.81)* | 6 (1.72)* | 5 (1.65)ᵃ | -4 (-1.13)* | 6 (2.38)ᵃ |

Note: ᵃWithin-group distinguishing statements significant at p<0.05.*Within-group distinguishing statements at p<0.01.

ᵇEE consensus statements non-significant at p>0.01. ᶜ EE consensus statements non-significant at >0.05.

ᵈER consensus statements non-significant at p>0.01. ᵉER consensus statements non-significant at p>0.05.
