# Supplementary figures and images for: Viewpoints on Factors for Successful Employment for Adults with Autism Spectrum Disorder
Source: PLoS One. 2015 Oct 13;10(10):e0139281. doi: 10.1371/journal.pone.0139281 (PMC4603894; doi:10.1371/journal.pone.0139281)

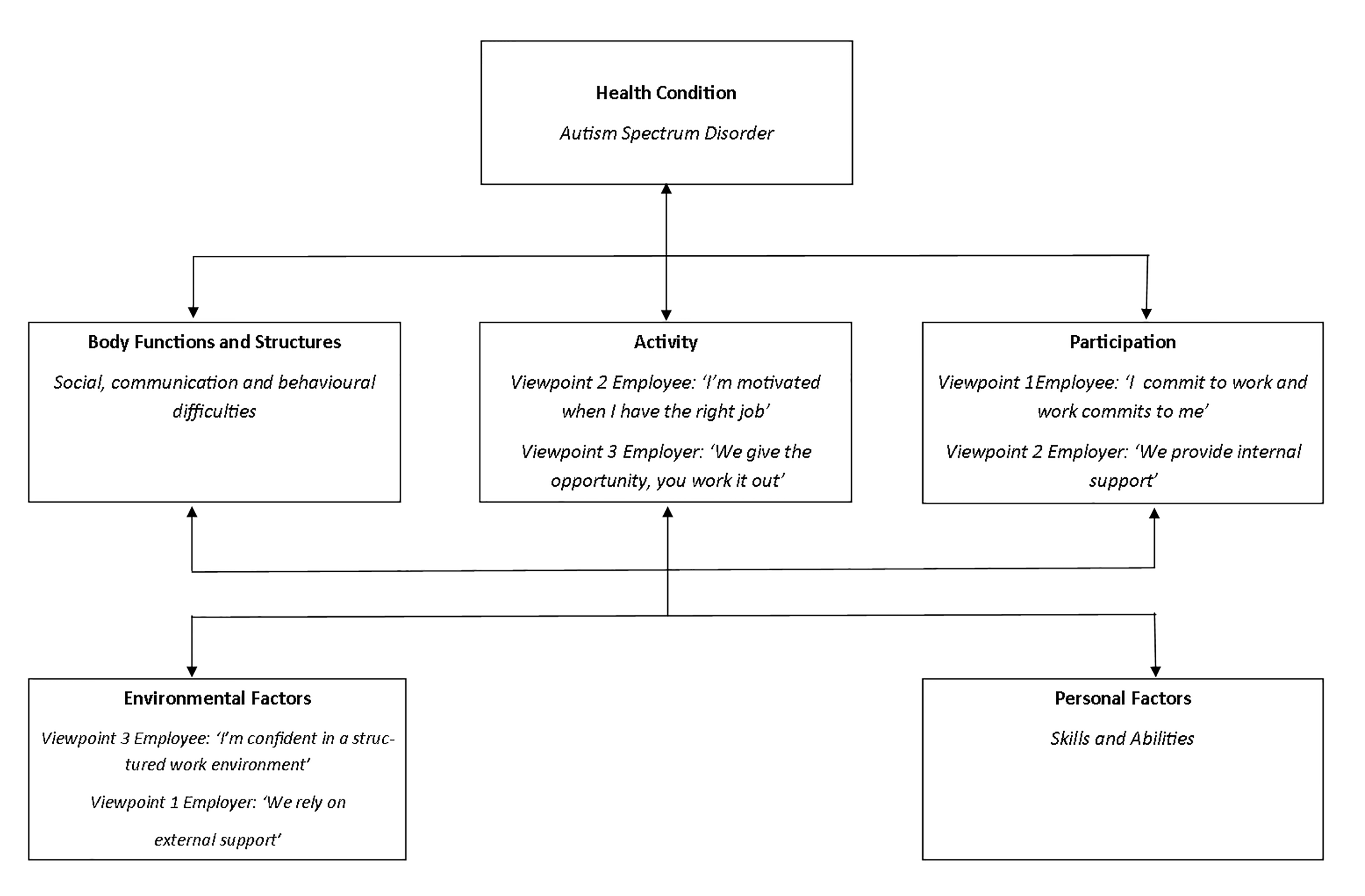

Supplement: S1 Fig — This figure is based on the World Health Organization ICF framework. (TIF) [file pone.0139281.s002.tif]
